# A qualitative study on barriers to utilisation of institutional delivery services in Moroto and Napak districts, Uganda: implications for programming

**DOI:** 10.1186/1471-2393-14-259

**Published:** 2014-08-04

**Authors:** Calistus Wilunda, Gianluca Quaglio, Giovanni Putoto, Peter Lochoro, Giovanni Dall’Oglio, Fabio Manenti, Andrea Atzori, Rose Miligan Lochiam, Risa Takahashi, Aline Mukundwa, Koyejo Oyerinde

**Affiliations:** Doctors with Africa CUAMM, via san Francesco 126, Padua, Italy; Department of Innovation, Research and Planning, Azienda ULSS 9, Treviso, Italy; Doctors with Africa CAUMM, P.O. Box 7214, Kampala, Uganda; College of life and Health Sciences, Chubu University, 1200 Matsumoto-cho, Kasugai, Aichi, Japan; Averting Maternal Death and Disability Program, Mailman School of Public Health, Columbia University, 60 Haven Avenue, B3, New York, NY 10032 USA

**Keywords:** Traditional birth attendants, Maternal health, Rural health, Pastoralist health care

## Abstract

**Background:**

Skilled attendance at delivery is critical in prevention of maternal deaths. However, many women in low- and middle-income countries still deliver without skilled assistance. This study was carried out to identify perceived barriers to utilisation of institutional delivery in two districts in Karamoja, Uganda.

**Methods:**

Data were collected through participatory rural appraisal (PRA) with 887 participants (459 women and 428 men) in 20 villages in Moroto and Napak districts. Data were analysed using deductive content analysis. Notes taken during PRA session were edited, triangulated and coded according to recurring issues. Additionally, participants used matrix ranking to express their perceived relative significance of the barriers identified.

**Results:**

The main barriers to utilisation of maternal health services were perceived to be: insecurity, poverty, socio-cultural factors, long distances to health facilities, lack of food at home and at health facilities, lack of supplies, drugs and basic infrastructure at health facilities, poor quality of care at health facilities, lack of participation in planning for health services and the ready availability of traditional birth attendants (TBAs). Factors related to economic and physical inaccessibility and lack of infrastructure, drugs and supplies at health facilities were highly ranked barriers to utilisation of institutional delivery.

**Conclusion:**

A comprehensive approach to increasing the utilisation of maternal health care services in Karamoja is needed. This should tackle both demand and supply side barriers using a multi-sectorial approach since the main barriers are outside the scope of the health sector. TBAs are still active in Karamoja and their role and influence on maternal health in this region cannot be ignored. A model for collaboration between skilled health workers and TBAs in order to increase institutional deliveries is needed.

## Background

Each year, about 1.1 million newborns and 179,000 mothers die in sub-Saharan Africa [1, 2]. Half of the world’s maternal, new-born, and child deaths occur in Sub-Saharan Africa, yet this geographic area has only 11% of the world’s population [3, 4]. The millennium development goals (MDGs) were adopted to support the improvement of social and economic conditions in the world’s poorest countries by 2015. While most countries have made some progress with some of the MDGs, the progress towards achieving MDG4 (reducing child mortality) and MDG5 (reducing maternal mortality and improving maternal health) has been uneven and the pace is too slow to meet set targets in most African countries [5]. Interventions to prevent maternal and newborn deaths are available and well known [6–9]. Of great significance in reduction of maternal and neonatal mortality is delivery care by a skilled provider [3, 10, 11]. Skilled attendance at delivery is arguably the single most important factor in preventing maternal deaths [10, 12]. However, many women in low- and middle-income countries still deliver outside health facilities for various reasons.

Determinants of utilisation of maternal care services have been widely investigated both qualitatively and quantitatively in different settings [10, 13, 14]. Whereas some determinants can be generalised, others are context specific [14, 15]. The practical factors influencing one behaviour are often different to those influencing another behaviour and the most effective interventions will be those directed at changing specific behaviours [10, 16]. Interventions for the reduction of perinatal and maternal mortality tend to focus on the skilled birth attendants and health facilities.

In Karamoja, the prospect of all births taking place within a health facility with skilled health personnel is still far from becoming reality; traditional birth attendants (TBAs) attend to a significant number of deliveries. Karamoja is a semi-arid and vulnerable region in North-East Uganda [17, 18]. The region has consistently demonstrated the nation’s lowest scores on key development and health indicators. In this region for example, coverage for skilled birth attendance and institutional delivery are 31% and 27% compared to the national averages of 58% and 57%, respectively [19]. Over 45% and 58% of men and women aged above 5 years have no formal education compared to the national averages of 12.5% and 20%, respectively [19]. Doctors with Africa CUAMM, an Italian non-governmental organisation, has been operating in Karamoja for about 30 years; working with district health offices and health facilities. The organisation has adopted the continuum of care approach as its main health service delivery strategy in its interventions [20].

Recent improvements in the policy environment in Uganda, rising socio-economic status and improvements in security have not resulted in robust increases in utilization of obstetric services at health facilities or a significant reduction in maternal deaths [21]. This study was carried out in order to identify barriers to utilisation of institutional delivery care services in Moroto and Napak districts in Karamoja.

## Methods

### Study area

Karamoja region, near the border with Kenya, occupies an area of 35,007 Km^2^ and has a population of 1,074,600. This study was conducted in April 2010 in Moroto and Napak districts. The two districts, with a total area of 8,516 Km^2^, had a population of about 270,650 in 2010. Although both districts are predominantly rural, Moroto District hosts Moroto town which has an urban/peri-urban population of about 11,600. Moroto town is the administrative headquarters of Karamoja Region and has a regional referral Hospital for the entire Karamoja. Most parts of Napak District have a flat terrain but parts of Moroto District are mountainous making them difficult to access even by car. Both districts are inhabited by Karamajong people whose main sources of livelihood are nomadic pastoralism and subsistence crop farming.

In Uganda, districts are subdivided into sub-counties, then parishes and villages. At the time of the study, Napak District had 6 sub-counties (Iriiri, Lokopo, Lopei, Lotome, Matany and Ngoloriet) and 200 villages whereas Moroto District had 5 sub-counties (Katikekile, Nadunget, Rupa, Northern Division and Southern Division) and 120 villages [22]. The districts had 61 nurses/midwives of different cadres, 11 doctors, 19 clinical officers and about 315 TBAs. In 2010 only 19% and 10% of deliveries took place in health facilities in Napak and Moroto districts, respectively [23], with most women delivering at home, attended to by either family members or TBAs. About 49% and 59% of the population in Moroto and Napak districts, respectively, is within five kilometres of a health facility. However, some of the health facilities are level II Health Centres which typically don’t offer maternity services. During rainy seasons most parts of the districts become inaccessible by motor vehicles due to muddy roads.

### Study population

**Figure 1:**
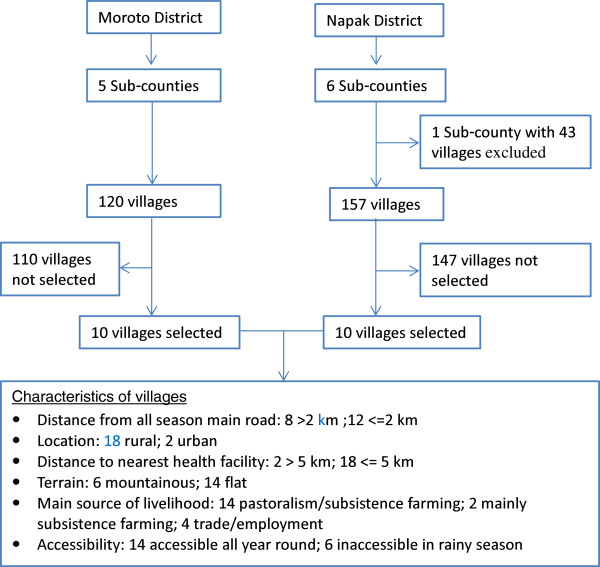
**Selection of Sub-counties and villages and characteristics of selected villages.**

The study was conducted in the catchment communities of health facilities in Moroto and Napak districts. These two districts were purposively selected because they were targets of a planned intervention to increase institutional delivery service by Doctors with Africa CUAMM. In consultation with district health authorities, twenty villages (ten in each district) located in 10 different sub-counties were selected purposively, to reflect the different geographic and socio-demographic characteristics of the communities in the districts. Figure 1 summarises the selection of villages and characteristics of the selected villages. In the selected villages, all women who had delivered in the past 5 years and their partners were eligible for the study.

### Study design and data collection

Data were collected through participatory rural appraisal (PRA). A total of 887 adult participants (459 women and 428 men) were recruited to participate in the PRA sessions. Participating villages were visited a day before the study and with the help of village leaders, potential participants were verbally invited to participate in the study the following day. The PRA data collection team consisted of a supervisor, two facilitators and two note takers. The supervisor and facilitators were experienced in PRA methodology having conducted similar studies in the same area in the past. The supervisor (co-author RML) further conducted a short training for facilitators and the note takers; covering the PRA methodology, the study objectives and a review of the tools. In order to overcome cultural factors that would limit freedom of expression, participants were divided into male and female groups. A male facilitator guided the male group while the female group was led by female facilitator. All members of data collection team were natives of the study districts and had a good understanding of the local culture and language. Two PRA sessions per group were held in each village and each session was made up of about 20 participants and lasted for about 3 hours. The sessions were held in public spaces selected by the communities, such as under trees and in local school buildings. Tools used during the sessions included community resource maps, Venn diagrams, matrix ranking, daily routines, and seasonal calendars. During the sessions, information on barriers to utilisation of maternal health services in the districts was collected using an open ended question guide which allowed for free discussion of the participants’ perceptions. During the discussions, the two note takers independently took notes. The discussions took place at alternate times for the male and female groups to allow the supervisor to attend both of them and also take notes. All notes were written in English as it was found to be easier to do so than to write in Karamojong (the local language). In case of lack of clarity, immediate clarification was sought. The main topics included in the question guide were: i) traditional practices and beliefs during delivery; ii) family support and decision making on health services during delivery; iii) the role of TBAs; iv) perceived quality of care and fee for services used; v) obstacles when using trained attendants’ services; vi) experience of delivery (including the services provided by the delivery attendants); and vii) reasons for a delivery outside a health care facility.

Matrix ranking was performed by asking participants to list main reasons why women in the village don’t deliver in health facilities. Participants were then asked to use stones to assign a score to each reason to reflect the relative weight of the reason in preventing women from using skilled birth attendants. One stone represented a weight of one. A literate member in the group facilitated the scoring exercise which was done by consensus among group members. Locally prepared refreshments were provided at the end of PRA sessions. No cash incentives were provided.

### Ethical considerations

This study was approved by the National Bioethics Committee at Uganda National Council for Science and Technology and by the Moroto District Health Management Team. Because most PRA participants were illiterate, and given that they participated in the study in groups, verbal informed consent was obtained from each PRA group after an explanation about the study.

### Data analysis

Data were analysed using deductive content analysis [24]. At the end of each PRA session, the study team reviewed, edited and harmonized the notes taken. They then read through the notes several times, triangulated the data collected from men and women groups, identified and coded all recurring issues by consensus and summarized them in a table. The issues were grouped under four themes in an adapted framework: (1) socio-cultural factors, (2) perceived benefit/need of skilled attendance, (3) economic inaccessibility and (4) physical inaccessibility [10]. The themes formed the framework for reporting. Scores from matrix ranking were summarized using a spider plot to reflect the perceived relative significance of each barrier identified by participants in preventing utilization of delivery services. Scores for each barrier at each site were rescaled to take values of 0–5 and then summed up.

## Results

**Figure 2:**
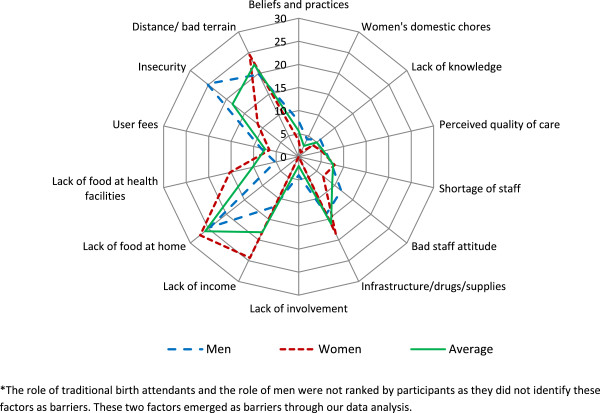
Relative scores of perceived barriers to utilization of delivery services among men and women in Moroto and Napak districts –scores obtained from matrix ranking*.

This study revealed a range of perceived barriers towards utilisation of institutional delivery services in Moroto and Napak districts. Table 1 presents a summary of the results and the details are presented below according to the four themes framework. The results of matrix ranking by study participants are presented in Figure 2. Women gave higher scores than men to lack of food at health facilities and lack of income. Men tended to give higher ranking than women to insecurity and bad staff attitude. Overall, factors related to economic and physical inaccessibility and lack of infrastructure, drugs and supplies at health facilities were the highly ranked barriers to utilisation of delivery care services. Only a few differences in the results were noted between the two districts: physical inaccessibility due to insecurity and bad terrain featured strongly in Moroto District whereas poor staff attitude and user fees came out more frequently in Napak District.

**Table 1:** **Barriers to utilisation of skilled delivery services in Moroto and Napak districts, Uganda**

Barrier	Findings
**I. Socio-cultural factors**	
Beliefs and practices	Cowards deliver at health units, beliefs related to disposal of placenta; cutting and tying of umbilical cord and expressing fear during delivery, delivering position, delivery is a private family issue, the ceremony of showing the baby to the public, the ceremony of naming a child, administration of traditional herbs
The role of men	Perceive maternal health as a women’s issue. Men are less emotionally and practically involved in maternal health
Women’s domestic chores	Nobody to prepare food at home and take care of children left behind while the woman is admitted
**II. Perceived benefit/need**	
Lack of knowledge	Lack on information about benefits of delivering in health units, low education status of women, lack of health education
Infrastructure, drugs and supplies	Lack of beds; light at night; drugs; supplies; equipment and water. Facilities not equipped to handle complications, few staffs to attend to women, long waiting time
Shortage of staff	
Perceived quality of care	
Bad staff attitude	Disrespectful staff, staff coming to work drunk or late, poor relationships between community and health staff, harsh treatment during delivery
Role of the TBAs	TBAs are acceptable, accessible, and affordable and offer a range of services. Confidence in trained TBAs. Women don’t deliver at health facilities unless they have been referred by TBAs.
Lack of involvement	Community not consulted about where to build health facilities, lack of information about facility catchment areas
**III. Economic inaccessibility**	
Lack of income	Poverty, costs of drugs and supplies, cost of ambulance, transportation costs
Lack for food at home	Lack of food at home for the family
Lack of food at health facilities	Lack of food for women delivering in health facilities and their caretakers
User fees	User fees at some health facilities
**IV. Physical inaccessibility**	
Insecurity	Inter-clan feuds and cattle rustling, insecurity at night, fear of the military
Distance/bad terrain	Long distance to health facilities, bad terrains, flooding of river valleys and muddy roads during rainy seasons
Lack of transportation means	Lack of reliable means of transportation even if women had money to pay for transportation

### Socio-cultural factors

Under this theme, the barriers identified were categorized as: i) traditional beliefs and practices, ii) the role of men and iii) women’s chores.

#### Traditional beliefs and practices

Herbs were believed to both prevent and treat a variety of problems during pregnancy and childbirth. They were believed to ease pain, to facilitate delivery and to avoid infection, as revealed in this quote:

*“There are local herbs to be administered for milk production and for stopping bleeding and it is inappropriate to give these herbs together with modern medicines” (*a woman in Loyaraboth village, Katikekile Sub-county, Moroto District)*.*

Some participants declared that they preferred to give birth at home because the placenta needed to be handled with care, since the disposal of it is related to traditional rituals and is associated with luck and misfortunes.

*“After pregnancy the placenta needs to be put in a certain location, to avoid evil spirits and bad omen”* (a man in Nakiloro village, Rupa Sub-county, Moroto District).

Participants also voiced cultural issues related to the handling of the umbilical cord. Tradition required that the umbilical cord of a baby girl be cut with a knife (the one used by women to prepare food), while the one of a baby boy is to be cut with an arrow (the one typically used by a man/warrior), but this could not be done in the health unit. It also emerged that the tying of the umbilical cord was a preserve of particular individuals as highlighted in this quote:

*“Only particular women of the village are allowed to tie the umbilical cord, with a special fibre from the bark of a tree, otherwise something wrong may happen. This is not possible in health facilities”* (a woman in Naoi village, Lopei Sub-county, Napak District).

The ceremony of bringing the newborn to the public is interfered with when a child is born at the health unit because the baby is exposed immediately after birth, and not kept in a particular house until the ceremony is done. It was believed this may bring misfortune. Some women said that they were used to their own traditional methods/positions of delivery (kneeling, squatting, sitting and lying on the side) which were disregarded in health facilities; therefore some of them preferred to deliver at home. Some women said the family perceived the health staff as strangers, as manifested in this citation:

*“Delivery is a family matter and mothers do not trust strangers with their babies” (*a woman in Natapar Apalemu village, Lotome Sub-county, Napak District).

It was believed that women should not show signs of fear during labour pains. But in case this happened in a health unit, external people would see it, delaying the delivery and bringing shame to the family. This perception is captured in this statement:

*“If the mother shows fear during delivery and this is noticed by the people of the village, this can delay the delivery and result into complications”* (a woman in Kobulin village, Iriir Sub-county, Napak District).

Child naming norms also emerged as a barrier to utilising institutional delivery. In some villages the ceremony of giving the right name to the baby involves elderly women well conversant with the names of the clan. Before the baby suckles for the first time, a list of names is mentioned, and the newborn takes the name mentioned at the very moment when the baby touches, with the mouth, the breast of the mother for the first time.

#### The role of men

Participants gave the impression that a man is the head of the household and the first decision maker. Nevertheless, maternal and child health care in the study districts was seen as a “women’s issue”. Men knew little about pregnancy and delivery, and were not fully involved. In several places, it emerged that, for the few women who make it to health facilities to deliver, the role of men was merely that of “sending” their partners to health facilities. The involvement of men appeared to be limited; both practically and emotionally. In some villages, men’s PRA groups demonstrated lack of emotional involvement by mentioning about the “cowardice” of women who fear to deliver at home and go to the health units.

*“Some coward women who cannot deliver by themselves go to that facility to be assisted to deliver”* (a man from Loyaraboth village, Katikekile Sub-county, Moroto District).

#### Women’s domestic chores

Women preferred to deliver at home to allow them to continue with family responsibilities like taking care of children and preparing meals. It was mentioned that women, especially those with other young children at home, disliked being admitted in health facilities because doing so caused suffering to children left at home with no one to take care of them, as highlighted in the statement below:

*“It is not good to leave other children alone at home with nobody to prepare food for them and go to stay in a health facility for two days to deliver. If you deliver at home you are able to continue to take care of your family”* (a woman in Kadilakieny village, Rupa Sub-county, Moroto District).

### Perceived benefit/need

The barriers identified under this theme were categorized as: i) lack of knowledge, ii) infrastructure, drugs, supplies staff shortage and perceived poor quality of care, iii) bad staff attitude, iv) the role of TBAs and v) lack of community involvement.

#### Lack of knowledge of the benefits of facility utilisation

Women did not deliver in health units because they didn’t know the benefits of doing so. The underlying cause of this problem was the low education status of women and a lack of health education on maternal health in their communities. Participants felt that there was a general lack of knowledge on maternal and child health issues in the communities in general and among women specifically.

#### Lack of infrastructure, drugs, supplies, staff and perceived poor quality

At some study sites, participants complained of a chronic lack of drugs and supplies at health facilities. As a result clients were always referred to private clinics and drug stores to buy drugs and supplies using their own money. This made them shy away from using health facilities. At some sites, women also complained of inadequacy of beds and beddings for mothers and children; a problem that often resulted into them sleeping on the floor. They also noted availability of only one delivery bed at health facilities resulting into problems in case two mothers are to be attended to at the same time as highlighted below.

*There are not enough beds for mothers and their babies in most health facilities. Our nearest facility has only one bed in the maternity unit. Supposing two women come to deliver at the same time, what will one of them use? The facility also doesn’t have enough blankets and something to cover the baby”* (a woman in Kobulin village, Iriir Sub-county, Napak District)

Lack of light at night was also cited as a reason for not utilizing health facilities especially if the mother had to stay overnight. Participants also noted lack of water supply at some health facilities. The quality of care at some health facilities was thus perceived to be poor. Besides lack of infrastructure, equipment and supplies, participants further noted that in some health facilities, there was only one staff to handle many patients. This was perceived to be an important factor that compromised the quality of care delivered, and increased waiting hours. Additionally, participants felt that some facilities were not well equipped to handle complicated deliveries.

#### Bad staff attitude

Bad staff attitude towards clients was mentioned in some communities as a reason for low utilization of health services in general and maternity services in particular. Some participants said their communities did not have a good relationship with staff of their health facility. They said health workers had a negative attitude towards them and did not attend to them with respect. Health workers were reported to be very rude which scared away mothers and made them to deliver at home. These quotes from two participants in different villages captures the issue of perceived bad staff attitude towards clients:

*“There is one health staff at our health facility who is rude, at times she beats mothers, and also refuses to give treatment to the patients”* (a man in Naoi village, Lopei Sub-county, Napak District).

*“The in-charge of the health unit is not cooperative with the patients and the staff. We have been appealing to the Government for the last five years, to transfer or to dismiss, but in vain…”* (a woman in Kobulin village, Iriir Sub-county, Napak District).

#### Role of the TBAs

The communities considered TBAs to be very important. Their services were accessed in the prenatal period and at the time of delivery. The participants said TBAs served multiple roles: giving advice especially to young women, sensitizing the community on maternal health issues, providing antenatal care, helping mothers at the time of delivery, administering local medicines and herbs as first aid since the health centre is far, massaging the pregnant women, referring complicated cases and escorting mothers and their newborns to health units for registration and immunizations. With TBAs being easily accessible, affordable and offering a range of services, they were preferred over health facilities in care seeking especially during delivery, as demonstrated by these statements:

*“We prefer TBAs, they are understanding and help us in many ways. If a woman is pregnant they visit and palpate the abdomen to see that the baby is fine and provide information on how to take care of the pregnancy. TBAs are also able to solve any problem that occurs during pregnancy. When labour begins a nearby TBA is called to help the woman to deliver. Sometimes when there is a problem, and she has tried and failed, she refers the woman to the health facility”* (a woman in Kadilakieny village, Rupa Sub-county, Moroto District).

*“TBAs are of great help to mothers especially those who deliver at night and cannot walk to the hospital due to insecurity and the long distance to the hospital”* (a woman in Kokeris village, Matany Sub-county, Napak District).

At most PRA sites, participants perceived that the role of TBAs was to attend to deliveries at home and refer mothers to health units for delivery only if a complication developed as revealed in this statement.

*“In our village, mothers do not use the nearby health facilities for delivery unless they are referred by TBAs”* (a woman in Lotorir village, Nadunget Sub-county, Moroto District).

However, further discussion on the services provided by TBAs brought up some challenges faced by these service providers, such as lack of equipment, lack of transport to refer complicated cases, limited knowledge, risk of infections, poor cleanliness and lack of motivation. In line with this, participants said that there was need to equip TBAs with necessary skills and supplies to enable them to perform their roles better as captured in this quote:

*“There is need for delivery kits to be given to TBAs because presently, they have only gloves and cotton”* (a woman in Kangolechin village, Ngoloriet Sub-county, Napak District).

Although the discussion on TBAs raised some criticisms, overall participants appreciated the role of TBAs and did not perceive them to be part of the problem and hence they did not include them in the matrix ranking (Figure 2).

#### Lack of community involvement

Participants in some areas felt that they were not involved in deciding where to locate health units. They felt that some of the current health facilities had been built with little consideration of where most people stayed. Some communities did not have an idea about the “health facility catchment areas” as always referred to by health workers. This was demonstrated by some participants wanting to have health units in their villages, despite having a health unit nearby.

### Economic inaccessibility

The barriers identified under economic inaccessibility were categorized as: i) lack of income, ii) lack for food at home, iii) lack of food at health facilities and iv) user fees.

The problems of lack of income, lack of food at home and at health facilities were ranked highly by women than men (Figure 2). Participants noted that some health facilities, the private-not-for-profit, charged fees for delivery services, and this affected the decision to deliver at those units. Although government health facilities were said to be free of charge, they had many hidden costs. Some health facilities required women to buy supplies such as cotton wool, soap, basins, clothes and polythene paper to use during or after delivery.

The issue of lack of food at health facilities for mothers admitted for delivery care came up at most PRA sites. At some facilities, mothers and their accompanying attendants were required to make their own feeding arrangements. With shortage of food at home, or nobody at home to prepare the food, it was difficult for women to feed during admission; additional feeding costs were incurred by women delivering in health facilities. The quote below highlights the problem of food in health facilities:

*“If you want to increase deliveries at the health facilities you have to distribute food for delivering mothers and their attendants”* (a woman in Kangolechin village, Ngoloriet Sub-county, Napak District).

The cost of the ambulance (20,000 Ugandan shillings, about 8 US $) was perceived to be too high, and well beyond the means of many families as expressed in the quote below:

*“We need an ambulance in Morulinga Health Centre to take us to Matany at a cheaper fee. We cannot afford the current fee they charge us for using the ambulance”* (a woman in Kokeris village, Matany Sub-county, Napak District).

Financial costs were exacerbated by high poverty levels and famine. Poverty was associated with lack of job opportunities to generate income and promote self-reliance. Participants went further to provide suggestions on how to solve the problem of poverty in the region such as provision of farm inputs to enable them to cultivate crops and support with setting up income generating activities.

### Physical inaccessibility

Regarding this theme, the barriers identified were categorized as: i) insecurity, ii) distance/bad terrain and iii) lack of transportation means.

#### Insecurity

Insecurity theme came up in all study sites and in both men and women PRA groups. Insecurity prevented mothers from attending deliveries in health units especially when labour began at night. Inter-clan feuds result in the displacement of some communities to areas that are far away from health units exacerbating the problem of geographical accessibility. During one of the PRA sessions in Naoi village, Lopei sub-county, Napak District, one woman gave a personal testimony of how she lost her baby due to insecurity.

*“The baby developed complications but I could not go to the health facility for security reasons. At that time there was an inter-clan feud. Women belonging to my clan were prevented from accessing care in the health unit because it is located in the other clan’s territory. Consequently, I lost my baby after delivery”.*Although the Ugandan government had deployed the army in the area to maintain security, the community felt terrified by the presence of the military. They said the army was more concerned with protecting livestock. Men gave a higher ranking to insecurity compared to women (Figure 2).

#### Distance, transportation and bad terrain

Long distance to health units, rough terrain and poor road network were cited as problems in accessing care at health facilities in most of the PRA sessions. Additionally, most health facilities did not have an ambulance to transport women during emergencies. The problem of terrain featured more in mountainous regions. Participants said it was very difficult for a mother to walk up and down the mountains during labour, putting her life and that of her baby at risk.

*“…expectant mothers with complications die before reaching the health unit on the other side of the mountain; there are no means of transport in this mountain, and the health unit is far…”* (A woman in Loyaraboth village, Katikekile Sub-county, Moroto District).

*“Please, finish constructing this nearby health unit to solve the problem of walking for a long distance to get to a health unit”* (a man in Lotirir village, Nadunget Sub-county, Moroto District where there is a HC II that does not offer normal delivery care).

The problem of rough terrain and poor road network became more severe during rainy season when roads in villages become impassable, and dry river beds become flooded making them impossible to cross. It was also noted that in some places even if women had money to pay for transportation, reliable means of transportation were lacking.

## Discussion

This study identified key perceived barriers to utilisation of institutional delivery care in Moroto and Napak districts in Uganda. Insecurity, lack of income, socio-cultural factors, long distances and poor terrain, lack of food at home and at health facilities, lack of basic infrastructure, drugs and supplies, availability of alternative providers (TBAs), perceptions of poor quality of care at health facilities and lack of participation in planning for health services were the main barriers to utilisation of delivery services. The problems of poverty and lack of food at health facilities were considered a major obstacle. Other reasons found were the trust and tradition that TBAs engendered; they shared the same culture and were long-serving members of the community. Our study found that home delivery was considered more convenient for some women because of their responsibilities to children or other household members. As observed by Kyomuhendo, in most communities in Uganda, the woman who delivers by herself is highly respected. On the contrary, those who deliver by caesarean section and those who die in childbirth are perceived to be weak. These perceptions partially help in understanding why women decide to deliver at home [21].

### Socio-cultural factors

This study confirms the implication of several beliefs recognized in previous studies in influencing choice of delivery site in Uganda [21, 25–27]. For example, women were reported to be shy about exposing their genitals during child birth and hence preferred squatting or kneeling [21, 26, 27]. Because these different delivery positions are not offered in health facilities in Uganda some women preferred to deliver at home. This is also the case in Moroto and Napak. Cultural sensitivity by health workers and accommodation, as much as possible, of the local cultural practices in service delivery could attract women to deliver in health facilities. For example, women could be allowed to deliver in their preferred birthing positions and the cultural practices related to the handling of umbilical cord and placenta by family members could be permitted at health facilities with modifications to ensure hygiene.

There is a growing awareness of the need to involve men in all stages of delivery. Men need to be targeted as key allies in improving institutional delivery utilisation [28, 29]. Current strategies in Uganda tend to focus mainly on women, yet it is men who provide financial support and frequently also make decisions to seek care [30]. Men in Napak and Moroto should be mobilised to participate in maternal health issues and provide support to their pregnant spouses for instance through existing social networks such as men’s groups, religious setups, and through public gatherings. The community needs to be involved in the future planning for maternal and neonatal health care services. It has been suggested that a community-based approach to dialogue among women, parents and communities to increase “demand” for institutional deliveries [30, 31], coupled with an improvement in the quality of delivery services, could improve attendance in Uganda, as it did for example in Nepal [32].

### Perceived benefit/need

A lack of awareness about the importance of skilled delivery attendants coupled with the non-recognition of the need for health services emerged from this study. Childbirth is often perceived in Uganda (and in other countries) as a normal event rather than an event which requires medical attention [13, 21, 33]. The lack of knowledge about danger signs can lead to delays in recognition of complications. Health promotion strategies can improve community awareness of the importance of skilled delivery attendance. This could be done through antenatal clinics [33, 34] since almost 97% of women in Karamoja attend at least 1 antenatal care visit [19], or community health education [35].

Considering the barriers of access to health facilities, it seems quite understandable that TBAs’ services are widely used. The high number of TBAs and the low number of professional health workers in the districts make TBAs easily accessible. As observed in other studies, TBAs’ kind and caring approach and their social and emotional closeness to the community, which creates loyalty and understanding, is highly appreciated [25, 33]. However, in 2009 the Ugandan Ministry of Health officially banned TBAs from conducting deliveries. After training in safe motherhood and referral management, willing TBAs were recruited into Village Health Teams (VHTs) [36]. The ban however has had little impact; many deliveries, mostly in rural areas, are still carried out by TBAs [37]. Among other impediments, a main obstacle of incorporating TBAs into VHTs is their loss of income. VHTs members are volunteers while TBAs are paid either monetarily or materially. So they cannot quit their ‘trade’ that easily.

In a recent meta-analysis of studies of deliveries assisted by TBAs, Wilson *et al.,* found that offering training, support, and resources to TBAs had a favourable impact on neonatal and perinatal mortality [38]; they provide evidence that trained and supported TBAs can contribute to reducing perinatal mortality. The authors recognized the heterogeneity of interventions in the included studies; however they argue that the consistency of the individual studies’ findings supports the message that TBAs make a difference [38, 39]. Considering that training of TBAs has a positive effect on perinatal outcomes, this strategy is worth reconsidering in Uganda, particularly in areas where health care facilities and personnel are still lacking and the utilization of TBAs is high. TBAs could be trained to support community-based care of the newborn and postpartum care of the mother, accompanied by a strong supervision by professional health workers. A study in Uganda has shown the benefit of training TBAs to refer cases of obstructed labour and fistulas but the authors note that for full efficacy of the TBA intervention, training must be accompanied by greater collaboration between health workers and TBAs [40]. Banning TBAs risks creating an operational pool of TBA practitioners without support of training and supervision and could break a linkage between the community and the formal health system.

The unwelcoming attitudes of health workers towards mothers may discourage institutional delivery. An intervention to promote better staff attitudes, such as using the patient-centred approach to care [41], and good interpersonal communication when dealing with service users [42] is warranted. Although change may be difficult to achieve, an intervention to improve the quality of maternity care could be rewarding, as observed in other studies in Uganda [25]. There is also need to promote regular dialogue between the community and the health workers to gather feedback on service delivery through for instance the health unit management committees. Lack of equipment, drugs, supplies and poor infrastructure can compromise both the perceived and the actual quality of care provided. The government and development partners need to increase resource allocation to ensure availability of these health system inputs in Moroto and Napak.

### Economic inaccessibility

Poverty appears to be a major factor influencing people’s decision-making about health services, as observed by other authors [33, 43, 44]. Since the main economic activities in Karamoja region is peasant farming and pastoralism, which have low earnings, the minimal costs related to institutional delivery were more likely to be unaffordable. Although user-fees were officially abolished in Uganda in 2001 [45], in practice some health facilities charge fees for delivery services. In some cases this is combined with additional costs for drugs and other medical supplies. There is need for the Ministry of Health to enforce the user fee policy through sufficient resource allocation and supervision. Women require more support in setting up income generating activities to improve household incomes. Experience from Bangladesh shows that women who are helped with loans from micro-credit programmes to start small income generating activities improve their household incomes and consequently their health care seeking behaviour including skilled birth attendance [46]. A deprived financial situation affects one’s ability to seek the most appropriate health care services [33, 44, 47].

### Physical inaccessibility

Poor road conditions and lack of transportation are associated with increased costs of visits to health care providers. This is a typical problem in remote rural areas in Africa. In Karamoja, this problem is aggravated by the unwillingness of women to make long trips at night, as a consequence of insecurity. Insecurity was ranked higher by men compared to women probably because besides preventing delivery service use, it directly affected men: they were more often targets of any insecurity crackdown by security agencies. In Uganda, rural communities are particularly affected by geographical barriers mainly because health facilities are mostly located in towns along main roads. A lot of progress to increase geographical accessibility by building more facilities has however been made in the country in the recent past with the proportion of households living within 5 km radius to a health facility having increased from 57% in 2000 [35] to 72% in 2010, and with a target of 80% by 2015 [48].

### Strengths and limitations

To the best of our knowledge this is the first ever published qualitative study from Karamoja region on barriers to institutional delivery. This study collected data from both men and women, and by triangulating the findings from the two groups, a comprehensive view of the perceived barriers to institutional delivery has been obtained. Additionally, purposive sampling ensured that the results obtained captured views of participants of different geographic and socio-demographic characteristics in the districts. This study has a number of limitations. The PRA sessions were not audio recorded as doing so would have inhibited free speech especially if it touched on insecurity related issues. Even though efforts were made to write down all important issues that arose during the discussions, some data might have been lost during the process. Data collectors however tried to minimize this, by reading back their notes to the participants and seeking clarification on unclear statements. Additionally, we did not use computer software to analyse the data and this might have led to loss of information. Despite these limitations, the findings of our study fit well into the main themes of barriers to institutional delivery use [10].

## Conclusions

This study has identified socio-cultural factors, perceived benefit/need, economic and physical inaccessibility issues as reasons why women don’t deliver at health facilities. Our findings suggest that: i) poverty alleviation strategies will contribute to improving access to and utilisation of maternal health care services; ii) the provision and maintenance of infrastructure may improve utilisation of maternal health care services, especially for communities living in remote areas; iii) health promotion programs to increase community awareness about safe delivery services may increase demand for maternal health services; iv) there is need to adopt a more inclusive approach to increase the accessibility of maternal health care services; v) in dealing with service users, health workers need to change their attitudes and improve interpersonal communication in a culturally sensitive manner; and vi) with many births taking place at home and with the high regard the community has for TBAs, involvement of TBAs in delivery of maternal health care is still important. Because they won’t disappear until every woman has access to a skilled health professional, there is need to define a model for a strong collaboration between professional health workers and TBAs in order to increase institutional delivery. The findings of this study provide local evidence that could help policy makers to develop strategies for the improvement of maternal and child health services.
